# Development and Initial Characterization of Cellular Models for COG Complex-Related CDG-II Diseases

**DOI:** 10.3389/fgene.2021.733048

**Published:** 2021-09-17

**Authors:** Farhana Taher Sumya, Irina D. Pokrovskaya, Vladimir Lupashin

**Affiliations:** Department of Physiology and Cell Biology, University of Arkansas for Medical Sciences, Little Rock, AR, United States

**Keywords:** COG complex, congenital disorder of glycosylation, Golgi apparatus, CRISPR, vesicle tethering, glycan processing, glycosylation, mass-spectrometry

## Abstract

Conserved Oligomeric Golgi (COG) is an octameric protein complex that orchestrates intra-Golgi trafficking of glycosylation enzymes. Over a hundred individuals with 31 different COG mutations have been identified until now. The cellular phenotypes and clinical presentations of COG-CDGs are heterogeneous, and patients primarily represent neurological, skeletal, and hepatic abnormalities. The establishment of a cellular COG disease model will benefit the molecular study of the disease, explaining the detailed sequence of the interplay between the COG complex and the trafficking machinery. Moreover, patient fibroblasts are not a good representative of all the organ systems and cell types that are affected by COG mutations. We developed and characterized cellular models for human COG4 mutations, specifically in RPE1 and HEK293T cell lines. Using a combination of CRISPR/Cas9 and lentiviral transduction technologies, both myc-tagged wild-type and mutant (G516R and R729W) COG4 proteins were expressed under the endogenous COG4 promoter. Constructed isogenic cell lines were comprehensively characterized using biochemical, microscopy (superresolution and electron), and proteomics approaches. The analysis revealed similar stability and localization of COG complex subunits, wild-type cell growth, and normal Golgi morphology in all three cell lines. Importantly, COG4-G516R cells demonstrated increased HPA-647 binding to the plasma membrane glycoconjugates, while COG4-R729W cells revealed high GNL-647 binding, indicating specific defects in O- and N-glycosylation. Both mutant cell lines express an elevated level of heparin sulfate proteoglycans. Moreover, a quantitative mass-spectrometry analysis of proteins secreted by COG-deficient cell lines revealed abnormal secretion of SIL1 and ERGIC-53 proteins by COG4-G516R cells. Interestingly, the clinical phenotype of patients with congenital mutations in the SIL1 gene (Marinesco-Sjogren syndrome) overlaps with the phenotype of COG4-G516R patients (Saul-Wilson syndrome). Our work is the first compressive study involving the creation of different COG mutations in different cell lines other than the patient’s fibroblast. It may help to address the underlying cause of the phenotypic defects leading to the discovery of a proper treatment guideline for COG-CDGs.

## Introduction

Golgi apparatus (GA) is the central organelle within the secretory pathway composed of flattened membranes organized in a cis medial-trans fashion (Huang and Wang, 2017; Makhoul et al., 2019). Cargo proteins and lipids go to the GA where they continue to undergo post-translational modification such as glycosylation. Thereafter, the cargo is sorted and trafficked to specialized compartments within the cell or is secreted. Golgi enzymes and cargo receptors constantly recycle within the Golgi (Derby and Gleeson, 2007; Bailey Blackburn et al., 2016). Multisubunit tethering complexes (MTCs) are the key components of the intracellular trafficking machinery that tether cargo containing transport vesicles and bring them close to their target membrane. They orchestrate trafficking events by setting up multiple interactions between their individual subunits with other components of the trafficking machinery namely SNAREs, SNARE-interacting proteins, Rabs, coiled-coil tethers, and vesicular coats (Ungar et al., 2006; Bröcker et al., 2010; Bailey Blackburn et al., 2016; D'Souza et al., 2020). The Conserved Oligomeric Golgi (COG) complex is a prominent MTC that mediates intra-Golgi retrograde trafficking by interacting with several cellular constituents of the vesicle docking/fusion machinery through specific interaction sites on its subunits (Bonifacino and Glick, 2004; Laufman et al., 2013; Willett et al., 2013c; D'Souza et al., 2020). Mutations in COG subunits result in a class of human diseases known as congenital disorders of glycosylation (CDG) type II (Ungar et al., 2006; D'Souza et al., 2020). COG-CDGs are defined as multi-systemic disorders with several distinguishable symptoms, including global developmental defects, dysmorphic features, microcephaly, and failure to thrive. These disorders are often accompanied by liver and neurological impairment (Foulquier, 2009; D'Souza et al., 2020; Ondruskova et al., 2021). Nowadays, one of the major issues related to CDGs is that many cases are either underdiagnosed or misdiagnosed.

Until now, over a hundred individuals with 31 different mutations in seven different COG subunits have been identified, presenting heterogeneous clinical features and cellular phenotypes. COG mutations cause a wide range of cellular defects that directly and indirectly impact glycosylation and other trafficking processes (Climer et al., 2018; D'Souza et al., 2020). Different types of mutations in each subunit of COG have been reported, which have been studied only in patients’ fibroblasts (Ferreira et al., 2018). Fibroblasts obtained from different patients could be very different in their genetic background and not a perfect representative of organ systems and cell types that are affected by COG mutations (D'Souza et al., 2020). To that end, a cellular COG disease model will benefit the molecular study of the disease, explaining the detailed sequence of the interplay between the COG complex and the trafficking machinery.

At first, we decided to develop a COG4-CDG disease model to facilitate an effective diagnosis and treatment. Two patients have been described with COG4-CDGIIj presenting clinical features such as developmental delay, hypotonia, failure to thrive, seizures, coagulopathy, liver cirrhosis, and recurrent infections. The second patient of this CDGII carried a C > T point mutation at position 2,185 in the COG4 gene resulting in R729W change (Reynders et al., 2009). Recently, a rare skeletal dysplasia has been discovered caused by a specific heterozygous COG4 substitution (p.G516R) named Saul-Wilson syndrome (SWS) (Ferreira et al., 2018; Zafra et al., 2021). Interestingly, symptoms exhibited by SWS patients are markedly different compared to COG4-CDGIIj individuals. The comparative study of two different types of COG4 mutation can potentially lead to a comprehensive understanding of the clinical phenotypes and the development of a working treatment protocol design. To create a cell-based model, we have chosen HEK293T (Stepanenko and Dmitrenko, 2015) and RPE1 as most of the COG-CDG patients exhibit neurological phenotypes as well as ocular defects (Climer et al., 2015, 2018; Blackburn et al., 2018). Moreover, these cells are non-cancerous in origin, obtain superior characteristics for microscopy imaging, and are easy to transfect as well as to study the genomic and functional properties.

In this study, we developed a cell-based COG4-CDGII disease model in RPE1 and HEK293T cell lines. Two different COG4 mutations have been introduced to benefit the molecular analysis of the disease, followed by the characterization of the developed isogenic cell lines for parameters essential for post-translational modification and regulatory secretion of the protein. We have applied biochemical, superresolution and electron microscopy, and proteomic approaches to accomplish the objectives of this study.

## Materials and Methods

### Cell Culture

hTERT RPE1 (Retinal Pigment Epithelial) and HEK293T cells (a human cell line obtained from embryonic kidney but exhibiting properties of immature neurons) purchased from ATCC were cultured in Dulbecco’s Modified Eagle’s Medium (DMEM) containing Nutrient mixture F-12 (Corning 10–092-CV) supplemented with 10% Fetal Bovine Serum (Atlas Biologicals, CF-0500-A). Cells were incubated in a 37°C incubator with 5% CO_2_ and 90% humidity.

### Creation of RPE1 COG4 Knockout Cell Line

RPE1-Cas9 stable cells (Khakurel et al., 2021) were transfected by the mixture of TransEDIT-dual plasmids (Transomic) with the following target sequences.

hCOG4 TransEDIT-dual 1 (TEDH-1017241-pCL/P-dua/-SFFV-ZsGreen).

grna-a: ACT​TTC​TCC​AAC​TCG​ACG​GCA​ACA​AGG​CTA

grna-b: CTG​TCA​GGG​AGC​GAA​TGA​GCT​CAG​CGG​AGA

hCOG4 transEDIT-dual 2 (TEDH-1017242-pCL/P-dua/-SFFV-ZsGreen)

grna-a: CAT​ATA​AGC​CAA​TTA​GCT​CCT​GCA​TGG​TAC

grna-b: CCA​AGA​TGT​CAT​CAG​CTC​TCT​GAA​TGG​CCT

Neon electroporation (Thermo Fisher) was used for transfecting cells according to the manufacturer’s protocol. 48 h after transfection, cells were collected by trypsinization, spun down at 600xg for 5 min, and resuspended in cell-sorting medium (PBS, 25 mM HEPES pH7.0, 2% FBS (heat-inactivated) 1 mM EDTA). Cell sorting was based on ZsGreen fluorescence; cells were sorted into a 96-well plate containing culture medium using a BD FACS Aria IIIu cell sorter. After sorting, colonies were expanded, and COG4KO phenotypes were confirmed by (Western Blot) WB analysis and (Immunofluorescence microscopy) IF for the absence of the COG4 protein.

### Introduction of Point Mutations (G516R and R729W) in COG4 cDNA and Construction of Plasmids

COG4-2xGFP in pEntra1A, was created by PCR of hCOG4-siRES-2xGFP-FRB (Willett et al., 2013b) and (BamHI/XhoI) subcloning into pEntra1A no ccDB (Campeau et al., 2009) (Addgene #17398) vector. COG4-G516R point mutation of C to G nucleotide (resulting in amino acid change G to R) was generated using Site-Directed Mutagenesis Kit (Agilent Technologies #200523) with primers 5'-CCA​GGA​CAT​CCA​GCG​CCG​GGT​GAC​AAG​TGC​CG-3' and 5' CGG​CAC​TTG​TCA​CCC​GGC​GCT​GGA​TGT​CCT​GG -3'. COG4-R729W point mutation of C to T nucleotide (resulting in amino acid R to W) was generated with primers 5'-CGA​GAC​AAG​TTT​GCC​TGG​CTC​TCC​CAG​ATG-3' and 5'- CAT​CTG​GGA​GAG​CCA​GGC​AAA​CTT​GTC​TCG -3'.

To confirm the COG4-G516R and R729W mutation, DNA sequencing has been done by UAMS DNA Sequencing Core using the following primers:

Primer 1: 5'- CTGGGCCCCAAATAATG -3'

Primer 2: 5'- TGTCCAGCAAAGTTCGTCAG -3'

Primer 3: 5'- TTC​TGT​TTG​AAG​GGA​TTG​CC -3'

Primer 4: 5'- GAC​ACC​TAT​GAG​AAG​GGC​CA -3'

Primer 5: 5'- ACG​GAG​CTC​AAC​AGC​ACA​G -3'

Primer 6: 5'- GGA​TAG​ACT​TCC​GCA​GTG​AA -3'

COG4-WT-3myc in pEntra1A, was created by EcoRI/XbaI subcloning of pSH-hCOG4-3myc (Whyte and Munro, 2001; Kudlyk et al., 2013) fragment into COG4-2xGFP in pEntra1A.

COG4-G516R-3myc in pEntra1A was created by BglII/Kpn1 subcloning of COG4-G516R-2xGFP in pEntra1A into COG4-WT-3myc in pEntra1A. COG4-R729W-3myc in pEntra1A was created by introducing the mutation (described above) in COG4-WT-3myc in pEntra1A.

To generate pLenti COG4 promoter Neo DEST construct, a chromosomal DNA fragment encoding the COG4 promoter region was amplified from human genomic DNA by PCR using the following forward and reverse primers:

Forward: 5'-GCTTATCGATTTCCCCCACGTCTGTTTACCA-3'Reverse: 5'- GAA​TTC​TAG​ACT​TGG​TCC​CCA​TTC​GGC​ACT​T -3'

Then, the amplified fragment was cloned into pLenti CMV Neo DEST (705-1) (Campeau et al., 2009) (Addgene 17292) or pLenti CMV Hygro (w117-1) (Addgene 17454), using XbalI and ClaI as restriction sites.

Plasmids were isolated from bacterial cells using the QIAprep Spin Miniprep Kit (Qiagen).

### Generation of Lentiviral Particles and Stable Cell Lines

At first, COG4-WT-3myc, COG4-G516R-3myc, COG4-R729W-3myc, COG4-WT-2xGFP, COG4-G516R-2xGFP in pEntra1A were recombined into pLenti COG4 promoter destination vectors using Gateway LR Clonase II Enzyme Mix (Thermo Fisher) according to the manufacturer’s instructions. HEK293FT cells were used for the generation of lentiviral particles. Three lentiviral packaging plasmids pMD2.G [a gift from Didier Trono (Addgene plasmid # 12259; http://n2t.net/addgene:12259; RRID: Addgene_12259)], pRSV-Rev, and pMDLg/pRRE (Dull et al., 1998), plus plasmid pLenti COG4 promoter DEST expressing different COG variants were used in equal amount. Then transfected HEK293FT cells were placed in serum-reduced Opti-MEM with 25 μM Chloroquine and GlutaMAX. The next day, the medium was changed to Opti-MEM supplemented with GlutaMAX. At 72 h after transfection, the medium was collected, and cell debris was removed by centrifugation at 600×g for 10 min. The supernatant was passed through 0.45 μM polyethersulfone (PES) membrane filter and the lentiviral medium was stored at 4°C overnight.

90% confluent RPE1 and HEK293T COG4KO cells growing in 6 well dishes were transduced with Lentiviral medium (200 µl per well). At 48 h after transduction of RPE1 COG4KO cells, the lentiviral medium was removed, and the cell growth media containing 600 µg/ml G418 was added for 48 h. The mixed rescued cells were expanded in media containing 200 µg/ml G418 and then cryopreserved in a freezing medium (90% FBS with 10% DMSO). In the rescued HEK293T cell line, the selection was made using 200 µg/ml Hygromycin for 48 h. The cells were expanded in media containing 50 µg/ml Hygromycin and then cryopreserved in a freezing medium (90% FBS with 10% DMSO).

### Secretion Assay

Cells were grown in 15-cm dishes to 90–100% confluency, rinsed 3 times with PBS, and placed in 20 ml serum-free, chemically defined medium (BioWhittaker Pro293a-CDM, Lonza) with GlutaMAX (Gibco). After 2 h, media was replaced with fresh serum-free media. 48 h later, the conditioned media was collected and clarified by 5 min centrifugation at 3,000xg to remove detached cells. The supernatant was collected and concentrated using a 10k concentrator (Amicon^®^ Ultra 10k, Millipore); the final concentration was 45× that of cell lysates. From these concentrated protein secretome samples (4 independent repeats for each analysis), the half amount was used for quantitative TMT mass spectrometry analysis, and the half amount was used for WB analysis.

### Preparation of Cell Lysates and Western Blot Analysis

To prepare the cell lysates, cells grown on tissue culture dishes were washed three times with PBS and lysed in hot 2% SDS. Samples were mixed with 6xSDS sample buffer containing *β*-mercaptoethanol and heated for 10 min at 70°C.

To prepare the protein secretome sample for WB analysis, 2× SDS sample buffer was added to the concentrated sample and heated at 95°C for 10 min 10–20 µg of protein was loaded into Bio-Rad (4–15%) or Genescript (8–16%) gradient gel. Proteins were transferred onto nitrocellulose membrane using the Thermo Scientific Pierce G2 Fast Blotter. Membranes were washed in PBS, blocked in Odyssey blocking buffer (LI-COR) for 20 min, and incubated with primary antibodies for 1 h at room temperature or overnight at 4°C. Membranes were washed with PBS and incubated with secondary fluorescently-tagged antibodies diluted in Odyssey blocking buffer for 1 h. Blots were then washed with PBS and imaged using the Odyssey Imaging System. Images were processed using the LI-COR Image Studio software.

### Lectin Blotting

To perform blots with fluorescent lectins, 20 µg of cell lysates or secretome samples were loaded onto Bio-Rad (4–15%) gradient gels, and proteins were transferred to nitrocellulose membrane using the Thermo Scientific Pierce G2 Fast Blotter. The membranes were blocked with Odyssey blocking buffer (LI-COR) for 20 min. Helix Pomatia Agglutinin (HPA)-Alexa 647 (Thermo Fisher) or Galanthus Nivalis Lectin (GNL) conjugated to Alexa 647 were diluted 1 : 1,000 in Odyssey blocking buffer from their stock concentration of 1 µg/µl and 5 µg/µl, respectively. Membranes were incubated with diluted HPA-Alexa 647 or GNL-Alexa 647 for 90 min and imaged using the Odyssey Imaging System.

### Heparin Sulfate Proteoglycans Analysis

The cells grown on 6-well tissue culture dishes to 100% confluency were detached with 10 mM EDTA. After that cell was centrifuged at 500xg for 5 min and washed with PBS twice. Each sample was treated with 200mU of Heparinase III in 100 µl of reaction buffer (100 mM NaCl, 20 mM Tris-HCL, and 1.5 mM CaCl_2_) for 1 hour at 37°C. Proteins from treated and untreated samples were solubilized in SDS sample buffer, resolved on 8–15% SDS-PAGE and immunoblotted with 3G10 monoclonal antibodies (AMSBio).

### Superresolution AiryScan Fluorescent Microscopy

Immunofluorescence microscopy was done using the previous protocol (Willett R. A. et al., 2013) with some additional modifications. Briefly, cells grown on 12-mm round coverslips to 80–90% confluency were fixed with paraformaldehyde (PFA, freshly made from 16% stock solution) diluted in phosphate-buffered saline (PBS) for 15 min at room temperature. For the lectin staining, 1% PFA was used for fixation, followed by incubations with 50 mM ammonium chloride for 5 min and two incubations in the blocking buffer (0.1% BSA in PBS). After that, cells were incubated with HPA-647 or GNL-647 diluted in blocking buffer for 30 min. For the antibody staining cells were fixed with 4% PFA, treated with 50 mM ammonium chloride (5 min), and permeabilized with 0.1% Triton X-100 (1 min) followed by two incubations with the blocking buffer. After 45 min incubation with primary antibodies diluted in the antibody buffer (1% cold fish gelatin, 0.1% saponin in PBS), cells were washed three times in PBS and incubated with fluorescently conjugated secondary antibodies diluted in antibody buffer for 30 min. Cells were washed four times with PBS, then coverslips were dipped in PBS and water 10 times each and mounted on glass microscope slides using Prolong^®^ Gold antifade reagent (Life Technologies). Cells were imaged with a 63× oil 1.4 numerical aperture (NA) objective of an LSM880 Zeiss Laser inverted microscope with Airyscan using ZEN software.

### Flow Cytometry

Cells grown to 80–100% confluency in a 6-well dish were detached using 10 mM EDTA and transferred into an Eppendorf tube. Cells were sedimented at 500xg for 3 min, washed with PBS twice, resuspended, and incubated for 30 min in ice-cold 0.1% BSA containing the lectin of choice (GNL-647, HPA-647) at a 1 : 500 dilution. Then ice-cold 0.1% BSA (plus DAPI for viability gaiting) was added and samples were analyzed using the NxT Attune flow cytometer. Cells were gated for live cells (DAPI excluding cells), singlets for correct cell size vs complexity. Analysis was done using FlowJo software**.**

### Mass Spectrometry (MS)

TMT mass spec analysis was performed at the UAMS IDeA National Resource for Quantitative Proteomics core. Proteins were reduced, alkylated, and purified by chloroform/methanol extraction prior to digestion with sequencing grade modified porcine trypsin (Promega). Tryptic peptides were labeled using tandem mass tag isobaric labeling reagents (Thermo) following the manufacturer’s instructions and combined into three 11-plex sample groups with a common reference sample. The labeled peptide multiplexes were separated into 46 fractions on a 100 × 1.0 mm Acquity BEH C18 column (Waters) using an Ultimate 3000 UHPLC system (Thermo) with a 50 min gradient from 98 : 2 to 60 : 40 buffer A : B ratio under basic pH conditions, and then consolidated into 18 super-fractions. Each super-fraction was then further separated by reverse-phase XSelect CSH C18 2.5 um resin (Waters) on an in-line 150 × 0.075 mm column using an UltiMate 3,000 RSLCnano system (Thermo). Peptides were eluted using a 60 min gradient from 98 : 2 to 60:40 buffer A : B ratio. Eluted peptides were ionized by electrospray (2.2 kV) followed by mass spectrometric analysis on an Orbitrap Eclipse Tribrid mass spectrometer (Thermo) using multi-notch MS3 parameters with real-time search enabled. MS data were acquired using the FTMS analyzer in top-speed profile mode at a resolution of 120,000 over a range of 375–1,500 m/z. Following CID activation with a normalized collision energy of 35.0, MS/MS data were acquired using the ion trap analyzer in centroid mode and normal mass range. Using synchronous precursor selection, up to 10 MS/MS precursors were selected for HCD activation with a normalized collision energy of 65.0, followed by the acquisition of MS3 reporter ion data using the FTMS analyzer in profile mode at a resolution of 50,000 over a range of 100–500 m/z.

Buffer A = 0.1% formic acid, 0.5% acetonitrile Buffer B = 0.1% formic acid, 99.9% acetonitrile. Both buffers adjusted to pH 10 with ammonium hydroxide for offline separation.

### Data Analysis (MS)

Protein TMT MS3 reporter ion intensity values are assessed for quality using our in-house ProteiNorm app, a user-friendly tool for a systematic evaluation of normalization methods, imputation of missing values, and comparisons of different differential abundance methods (Graw et al., 2021). Popular normalization methods are evaluated, including log2 normalization (Log2), median normalization (Median), mean normalization (Mean), variance stabilizing normalization (VSN) (Huber et al., 2003), quantile normalization (Quantile) (Bolstad et al., 2010), cyclic loess normalization (Cyclic Loess) (Ritchie et al., 2015), global robust linear regression normalization (RLR) (Chawade et al., 2014), and global intensity normalization (Global Intensity) (Chawade et al., 2014). The individual performance of each method can be evaluated by comparing the following metrices: total intensity, Pooled intragroup Coefficient of Variation (PCV), Pooled intragroup Median Absolute Deviation (PMAD), Pooled intragroup Estimate of Variance (PEV), intragroup correlation, sample correlation heatmap (Pearson), and log2-ratio distributions. The normalized data was used to perform statistical analysis using Linear Models for Microarray Data (limma) with empirical Bayes (eBayes) smoothing to the standard errors (Ritchie et al., 2015). We performed limma differential abundance analysis using a paired sample design to evaluate differences between injured and naïve samples. Proteins with an FDR adjusted *p*-value <0.05 and a fold change >2 are considered significant. Significant proteins were utilized to identify important protein networks and pathways using the Ensemble of Gene Set Enrichment Analyses (EGSEA) Bioconductor package and Qiagen’s Ingenuity Pathway Analysis (Alhamdoosh et al., 2017).

### Transmission Electron Microscopy

Samples were treated according to Valdivia’s lab protocol (Cocchiaro et al., 2008) with some modifications (Pokrovskaya et al., 2012). In short, cells were fixed for 20 min on ice with 2.5% glutaraldehyde and 0.05% malachite green (EMS) in 0.1 M sodium cacodylate buffer, pH 6.8. Samples were post-fixed for 30 min at room temperature with 0.5% osmium tetroxide and 0.8% potassium ferricyanide in 0.1 M sodium cacodylate, for 20 min on ice in 1% tannic acid, and for 1 h in 1% uranyl acetate at room temperature. Specimens were dehydrated with a graded ethanol series and embedded in Araldite 502/Embed 812 resins (EMS). Ultrathin sections were imaged at 80 kV on FEI Technai G2 TF20 transmission electron microscope. Digital images were acquired with FEI Eagle 4kX USB Digital Camera.

### Antibodies

Primary and secondary antibodies used for WB or IF were commercially purchased. The list of antibodies and their dilutions are described in Supplementary Table S2.

### Statistical Analysis

All the WB images are representative of 3 repeats and those were quantified by densitometry using the LI-COR Image Studio software. Error bars for all graphs represent standard deviation. Statistical analysis was done using one-way ANOVA using GraphPad Prism software.

## Results

### Expression of COG4-G516R and COG4-R729W Mutant Proteins Rescued the Major COG Knockout Phenotypes

**FIGURE 1:**
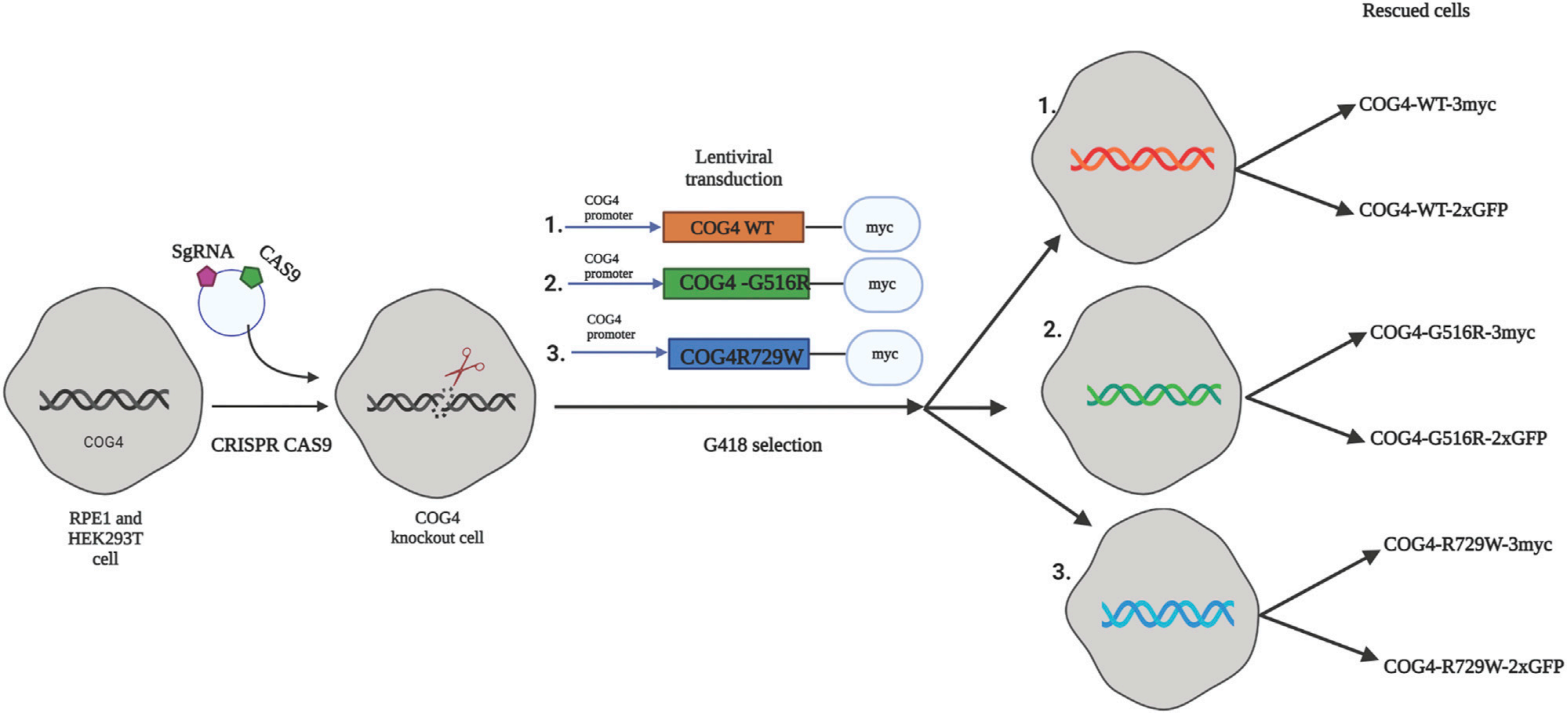
The strategy for the creation of cellular COG-CDG models. A conceptual design of the COG4-SWS and COG4-CDGIIj cellular models. In the first step CRISPR/Cas9 protocol was used to make RPE1 and HEK293T COG4 KO cells lines. On the second step, lentiviral transduction and G418 selection was utilized to create populations of isogenic cells expressing GFP or 3myc-tagged COG4 variants under endogenous COG4 promoter.

As a general strategy for the creation of mutant cell lines for functional and proteomics studies, we first knocked out the endogenous COG4 by CRISPR/Cas9 protocol in both HEK293T (Blackburn and Lupashin, 2016) and RPE1 cells and then re-introduced tagged wild-type or mutant variants of COG4 expressed under the control of the endogenous COG4 promoter (Figure 1). As a result, we developed a set of rescued RPE1 and HEK293T cell lines expressing COG4 (WT/G516R/R729W)-2xGFP and COG4 (WT/G516R/R729W)-3myc. Preliminary data obtained with GFP-tagged COG4 indicated that all three variants were expressed to near endogenous levels, Golgi localized, and able to rescue many COG4 KO trafficking and glycosylation defects with the exception of Cathepsin D sorting and TMEM165 stability (data not shown). We concluded that the addition of the bulky 2xGFP tag is partially compromising COG4 function and therefore focused our investigation on the COG4 constructs tagged with 3myc.

**FIGURE 2:**
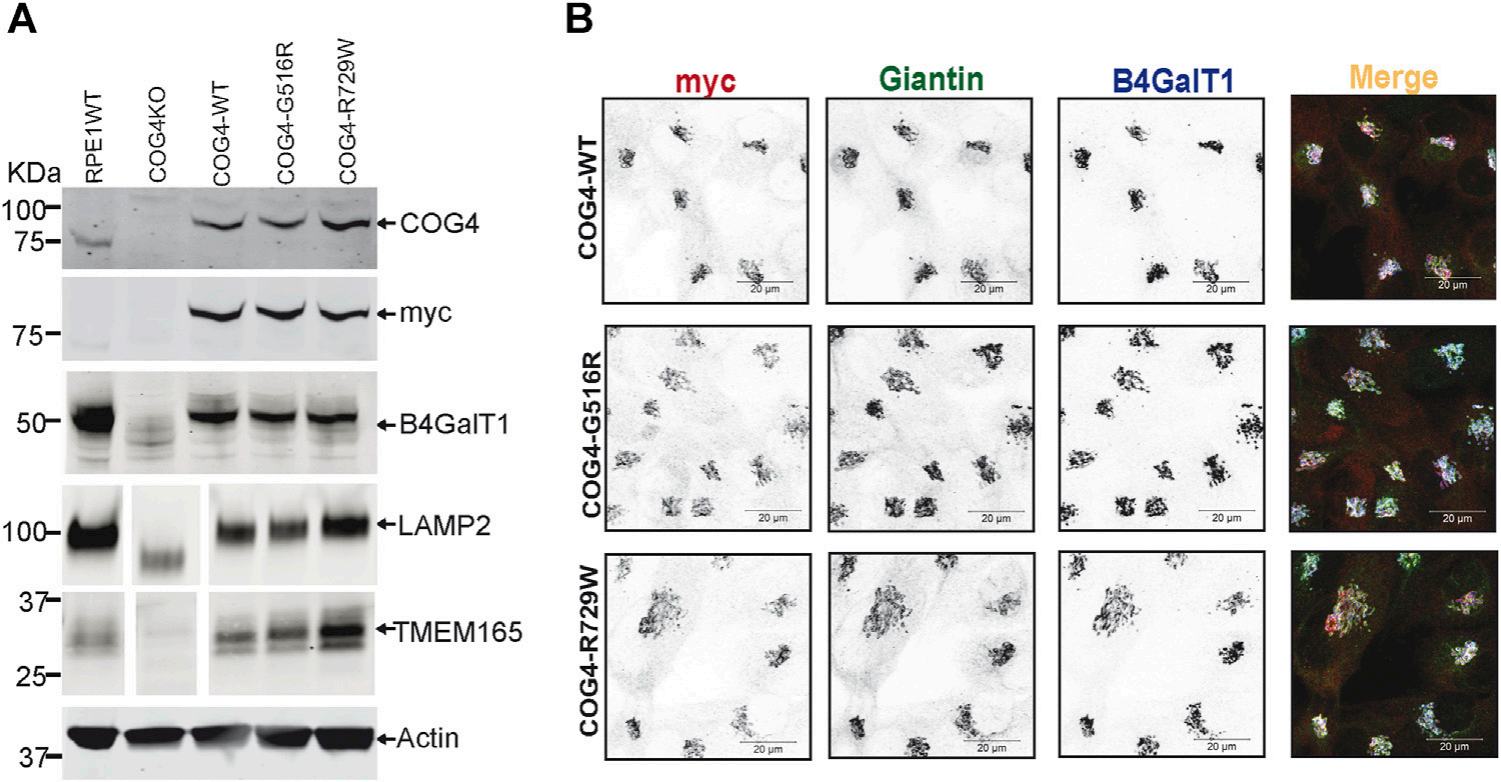
Expression of G516R and R729W mutant proteins rescues major cellular phenotypes associated with COG4 deficiency. **(A)** WB to detect COG4, myc, B4GalT1, Lamp2, Tmem165, and actin in total cellular lysates prepared from RPE1 wild type, COG4 KO, rescued COG4-WT-3myc, COG4-G516-3myc, and COG4-R729W-3myc cells. Actin has been used as a loading control. **(B)** Airyscan superresolution IF analysis of RPE1 cells stained for myc (red), Giantin (green), B4GalT1 (blue). The red, green and infrared (blue) channels are presented in inverted black and white mode, whereas the merged view is shown in RGB mode. Scale bars, 20 µm.

To characterize the rescued cell lines, wild-type and mutant cells have been analyzed using the biochemical and IF approaches (Figures 2A,B, Supplementary Figure S1A,B). WB analysis of the total protein revealed that the COG4 expression levels are similar in all three rescued RPE1, HEK293T cell lines and very close to the expression level of the endogenous COG4 in RPE1 and HEK293T cells, respectively, indicating that our strategy indeed resulted in the near-endogenous level of COG4-3myc expression (Figure 2A, Supplementary Figure S1A). Analysis of the myc signal confirmed a similar level of expression of all three COG4 variants, indicating that neither G516R nor R729R mutations affect the stability of COG4 protein. This result contrasts with the data previously published for COG4-G516R mutant fibroblasts (Ferreira et al., 2018). Superresolution microscopy has demonstrated that the myc tagged COG4 is Golgi localized in all rescued cell lines as myc signal colocalized with Giantin (cis-Golgi protein) and B4GalT1(trans-Golgi enzyme) (Figure 2B). Similarly, myc tagged COG4 is Golgi localized in all rescued HEK293T cell lines as myc signal is colocalized with GM130 (Supplementary Figure S1B). All analyzed cells demonstrated a similar COG4-3myc staining pattern, indicating that the rescued cell population is uniform and that cellular localization of COG4 mutants is indistinguishable from the localization of wild-type protein. Previously, we have reported that the Golgi enzyme stability and glycosylation of Lamp2 (Lysosomal membrane protein) and TMEM165 (a Golgi transmembrane glycoprotein) are altered in COG knockout cells. Western blot analysis of COG4 depleted HEK293T (Bailey Blackburn et al., 2016) and RPE1 cells show an increase in the electrophoretic mobility of Lamp2 and TMEM165 and depletion of B4GalT1enzyme (Figure 2A, Supplementary Figure S1A)**.** Expression of wild-type and mutant COG4 rescued stability and glycosylation of all three tested proteins (Figure 2A, Supplementary Figure S1A). IF analysis of the mutant cell line also revealed no depletion or mislocalization of B4GalT1 or COG-sensitive protein Giantin (Figure 2B), indicating that both wild-type and a mutant version of COG4 can rescue major biochemical phenotypes associated with COG4 depletion.

### Golgi Morphology Is Not Altered in Cells Expressing COG4 G516R and R729W Mutant Proteins

**FIGURE 3:**
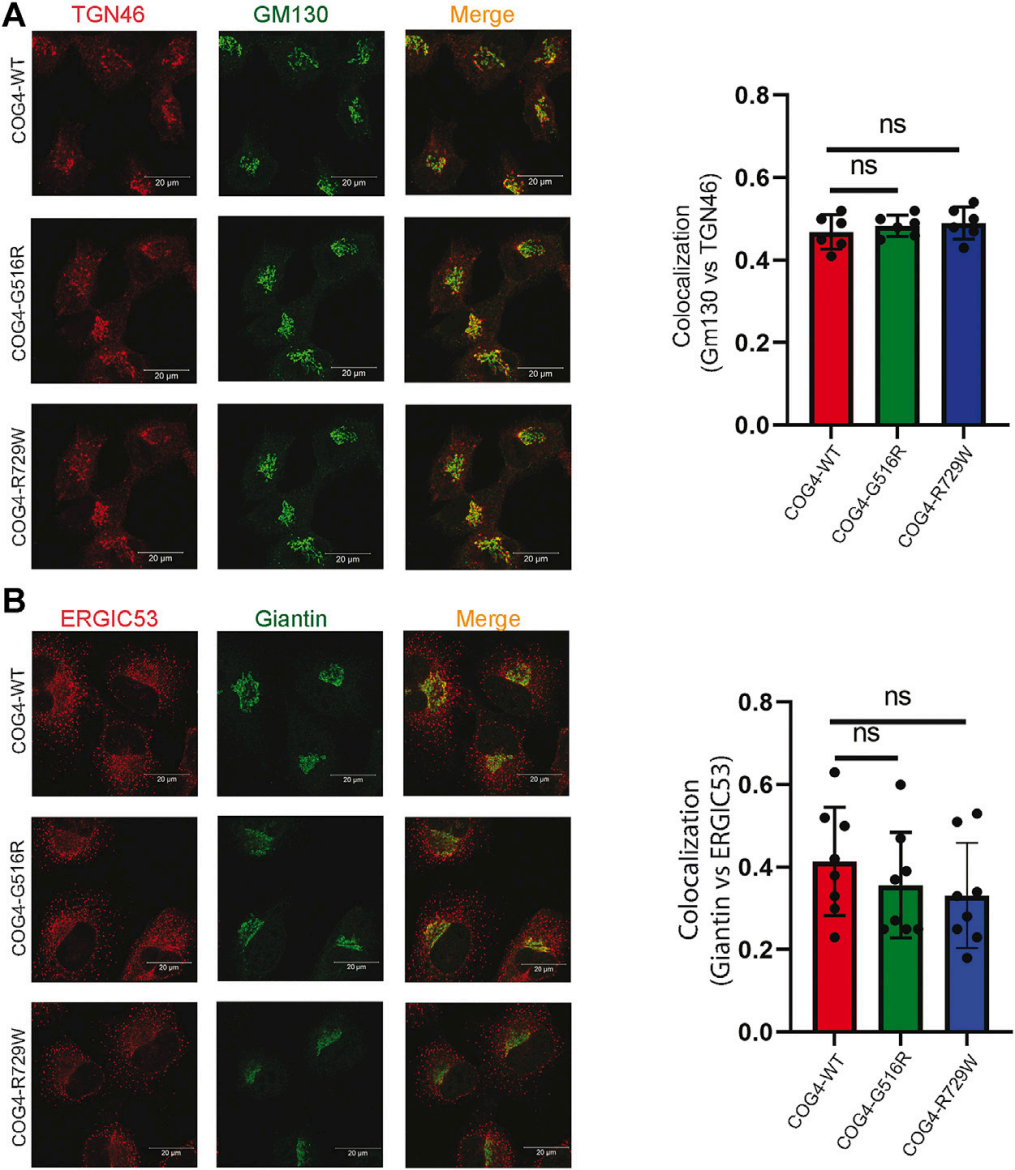
Expression of COG4 mutants does not alter Golgi morphology. (A) Airyscan superresolution IF analysis of RPE1 COG4 wild type, G516R and R729W rescued cells stained for **(A)** GM130 (green) and TGN46 (red) and **(B)** GM130 (green) and ERGIC53 (red). Scale bars, 20 µm. Colocalization of TGN46 vs GM130 and ERGIC53 vs GM130 has been performed using Pearson’s correlation coefficient (Right panel for both **(A)** and **(B)**, n = 20. Statistical significance was calculated by GraphPad Prism 8 using one-way ANOVA. *p* > 0.05, non-significant (ns). Error bar represents mean ± SD.

Previous studies suggested that COG4 mutations affect the overall structure of the Golgi apparatus. Golgi morphology was significantly altered in the COG4-G516R patient’s fibroblast, which was determined by colocalization of GM130 and TGN46 (suggesting the collapse of the *cis*- and trans-Golgi stacks) (Ferreira et al., 2018). Moreover, undulated, fragmented, and disrupted Golgi has been observed in the COG4-R729W mutant patient’s fibroblast (Reynders et al., 2009). Guided by the findings from these studies, we characterized the RPE1 COG4 mutated cell line for Golgi morphology. We applied the superresolution IF approach to check the colocalization of GM130 (peripheral membrane protein of cis-Golgi) and TGN46 (a membrane protein localized in trans-Golgi network) in the rescued mutant cell lines (both COG4-G516R and COG4-R729W). The result revealed no significant difference in relative colocalization of cis and trans-Golgi markers in comparison with the cells rescued with wild-type COG4-3myc (Figure 3A)**,** indicating no collapse of *cis*- and trans-Golgi in mutant cell lines. In addition, colocalization analysis of ERGIC53 (membrane protein of the endoplasmic reticulum-Golgi intermediate compartment) and Giantin (medial-Golgi protein) revealed no significant alteration in colocalization of those markers in both mutated cell lines compared to wild type (Figure 3B). IF analysis of HEK293T cell lines confirms that Golgi morphology was undisturbed in cells expressing COG4 point mutants (Supplementary Figure S2A,B); moreover, EELS, the hallmark of COG deficiency in HEK293T cells (D'Souza et al., 2019) was not accumulated in cells expressing either G516R or R729W COG4 mutants (data not shown).

**FIGURE 4:**
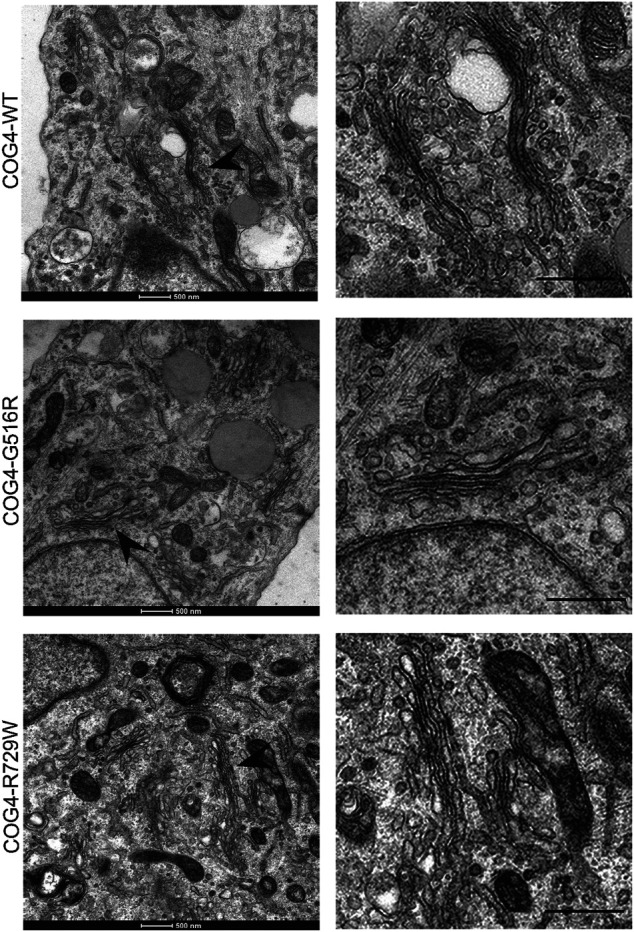
Golgi ultrastructure is not perturbed in cells expressing COG4 mutants. Transmission Electron Microscopy has been performed to see the detailed ultrastructure of rescued RPE1 COG4-WT-3myc, COG4-G516R-3myc, and COG4-R729W-3myc cell lines. Black arrowhead indicates the Golgi. On the right side, the zoom view of the Golgi region is shown. The scale bar is 500 nm.

Since the resolution of the Airyscan microscopy (160 nm in x-y dimension) may not be sufficient to detect specific changes in Golgi morphology, we have employed TEM (Transmission Electron Microscopy) to analyze Golgi structure in cells bearing COG4 mutant proteins. The analysis revealed the Golgi stacks morphology and integrity were normal in all analyzed cell lines (Figure 4). These results suggest that altered Golgi morphology observed in patient’s fibroblasts may not be directly linked to point mutations in COG4.

### COG4 Mutations do Not Affect the Localization of V-SNARE GS15

**FIGURE 5:**
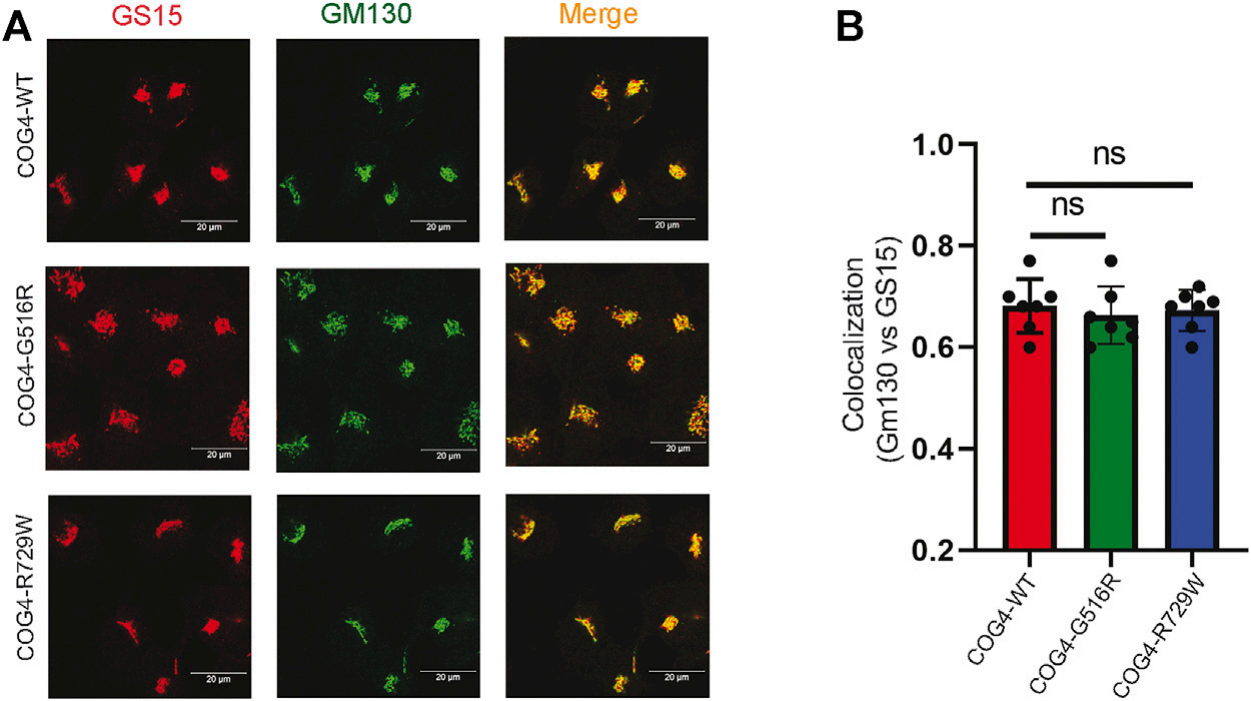
Expression of G516R and R729W mutants do not affect the localization of v-SNARE GS15. **(A)** RPE1 rescued COG4-WT-3myc, COG4-G516R-3myc, and COG4-R729-3myc cells were stained with GM130 (green) and GS15 (red). Scale bar 20um. **(B)** Colocalization of GS15 with GM130 has been analyzed using Pearson’s correlation coefficient. Quantification of colocalization in 20 cells analyzed. Statistical significance was calculated by GraphPad Prism 8 using one-way ANOVA. *p* > 0.05, nonsignificant (ns). Error bar represents mean ± SD.

GS15/Bet1L is a known COG-sensitive Golgi SNARE protein (Oka et al., 2004; Laufman et al., 2013; Blackburn et al., 2019). In the COG7-deficient patient’s fibroblast, the Golgi staining for GS15 protein was substantially reduced and more dispersed as compared to control fibroblasts (Steet and Kornfeld, 2006). We were interested to see if the COG4 mutations are affecting the GS15 localization or not. IF analysis has been performed by staining the rescued COG4 (G516R and R729W) mutant and COG4WT cell lines with GM130 and GS15. The superresolution confocal microscopy revealed no significant colocalization difference of GM130 and GS15 in both mutants in comparison to wild type. In addition, the intensity of the GS15 signal was not altered in the mutant (Figures 5A,B). This result indicates the investigated COG4 mutations do not significantly affect GS15 localization or stability.

### COG4-G516R and COG4-R729W Mutations Cause O-Glycosylation and N-Glycosylation Defects, Respectively

**FIGURE 6:**
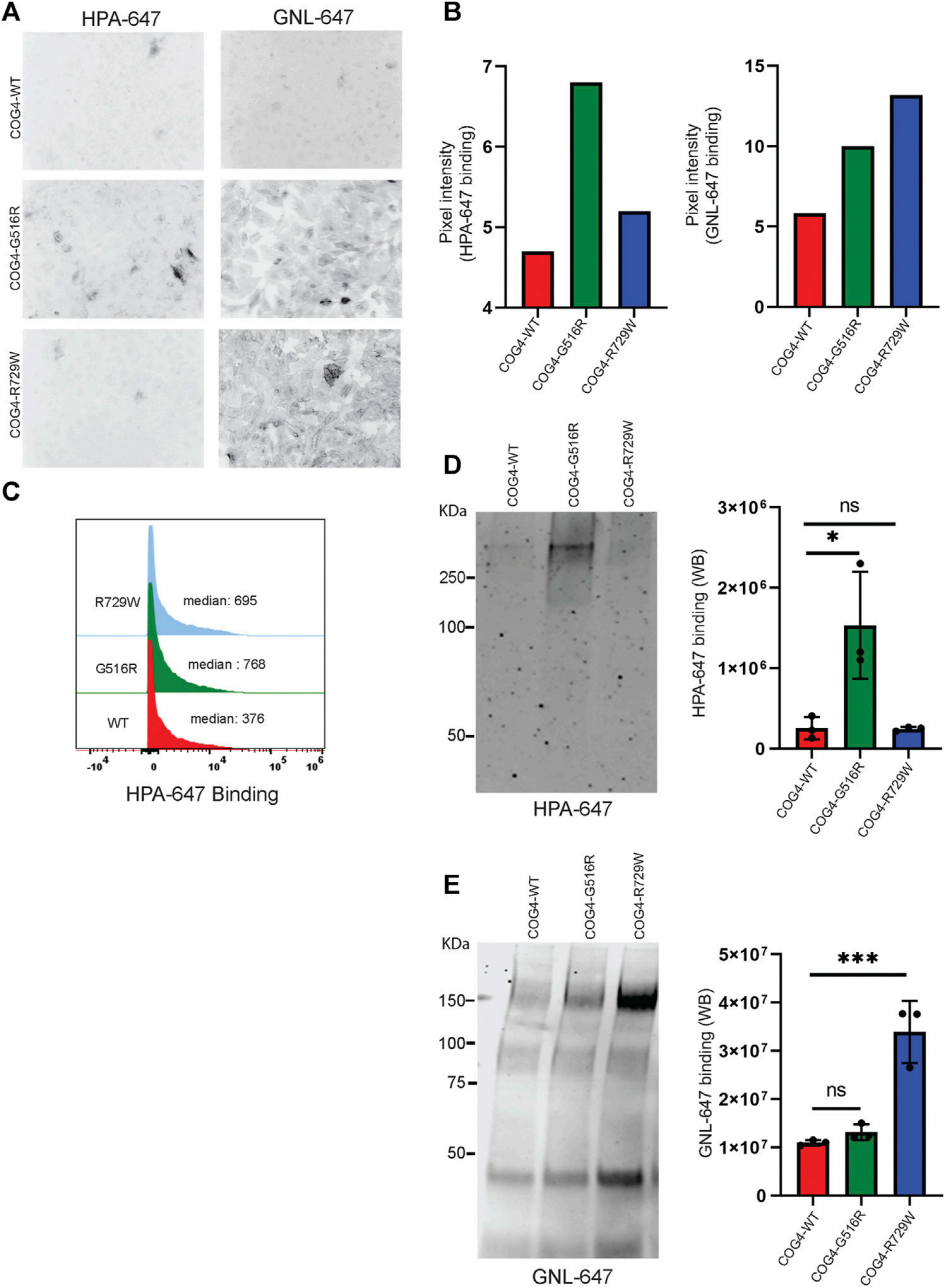
COG4-G516R and COG4-R729W mutations are causing O-glycosylation and N-glycosylation defects, respectively**. (A)** Non-permeabilized RPE1 rescued COG4-WT-3myc, COG4-G516R-3myc, and COG4-R729W-3myc cells were stained with the fluorescently conjugated lectins HPA-647 (specific for terminal GalNAc residues) and GNL-647 (specific for terminal α-D-mannosyl residues). The images were taken by Zeiss Axiovert 200M fluorescent microscope using a ×20 objective. **(B)** The pixel intensity of the full image for each cell line has been measured using ImageJ software. The bar graph has been presented with statistical value to provide intensity difference for each lectin (HPA-647, GNL-647) binding among the rescued cell line. **(C)** Flow cytometry histogram of HPA-647 fluorescence intensity in COG4-WT-3myc (red), COG4-G516R-3myc (green), and COG4-R729W-3myc (blue) population after gating for live and single cells. **(D)** Western blot analysis of the secretion from the rescued cells has been performed by probing with the HPA-647 antibody **(left panel).** Quantification of the lectin HPA-647 binding intensity **(Right panel).** Statistical significance was calculated by GraphPad Prism 8 using One-way Anova. *p ≤ 0.05, **p ≤ 0.01 and non-significant (ns) *p* > 0.05. Error bar represents mean ± SD. **(E)** Western blot analysis of the secretion from the rescued cells has been performed by probing with the GNL-647 antibody **(left panel).** Quantification of the lectin GNL-647 binding intensity **(Right panel).** Statistical significance was calculated by GraphPad Prism 8 using One-way ANOVA. *p ≤ 0.05, **p ≤ 0.01 and non-significant (ns) *p* > 0.05. Error bar represents mean ± SD.

After finding that both G516R and R729W COG4 mutant proteins are capable of performing the major COG4 functions, we asked the question – what is altered in cells expressing COG4 point mutants? Previous studies reported that COG complex subunit knockdowns (KD) cause altered binding of several lectins due to impaired glycosylation of plasma membrane glycoconjugates (Shestakova et al., 2007; Richardson et al., 2009; Pokrovskaya et al., 2011; Climer et al., 2018). *Galanthus nivalus* lectin GNL binds to terminal α1-3 linked mannose residues exposed on immature N-glycosylated plasma membrane glycoproteins in all tested COG KD and KO cells (Pokrovskaya et al., 2011; Bailey Blackburn et al., 2016). Also, another lectin HPA (*Helix Pomatia* Agglutinin), binds to terminal N-acetylgalactosaminyl residues in immature O-glycosylated glycoproteins (Brooks, 2000). We have utilized GNL, and HPA conjugated with Alexa-647 (GNL-647 and HPA-647) to check the 'N' and 'O'-glycosylation fidelity in COG4-G516R and COG4-R729W cell lines in IF, WB, and flow cytometry approaches (Figure 6). IF experiment revealed that binding of HPA was significantly increased to the plasma membrane of cells expressing COG4-G516R (Figures 6A,B, Supplementary Figure S3A). The flow cytometry also confirmed that HPA-647 binding increased in cells expressing mutant COG4 (Figure 6C). In contrast, binding of GNL to plasma membrane of non-permeabilized cells was increased in both mutant cell lines, but most significantly in cells expressing COG4-R729W (Figures 6A,B, Supplementary Figure S3B). This lectin data is in good agreement with previously published results obtained with fibroblasts isolated from COG4-CDG (CDG-IIj) (Reynders et al., 2009) and COG4-SWS (Ferreira et al., 2018) patients. In addition, WB lectin analysis of secreted glycoproteins also revealed that HPA-647 and GNL-647 binding were significantly increased in G516R and R729W mutants correspondingly (Figures 6D,E).

The combined result indicates a notable O-glycosylation defect in cells expressing COG4-G516R and N-glycosylation defect in cells expressing COG4-R729W mutants.

### Increased Accumulation of Core Proteins of HSPGs (Heparin Sulfate Proteoglycans) on the Cell Surface of COG4 Mutants

**FIGURE 7:**
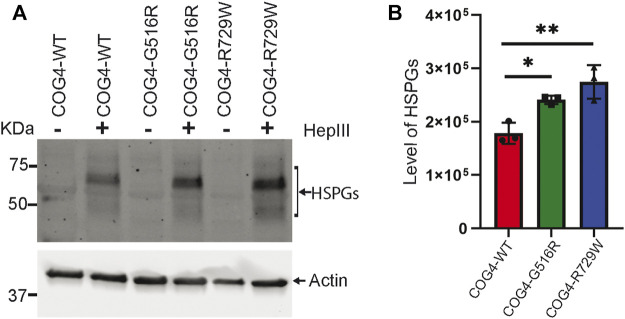
Increased accumulation of core proteins of HSPGs (Heparin sulfate proteoglycans) on the cell surface of rescued mutant (G516R and R729W) RPE1 cell line. **(A)** RPE1 rescued COG4-WT-3myc, COG4-G516R-3myc, and COG4-R729W-3myc cells were digested by the Heparinase III enzyme. Western blot analysis with 3G10 antibody to the core proteins of proteoglycan (HSPGs). **(B)** Quantitative analysis of the core proteins of HSPGs accumulation. Statistical significance was calculated by GraphPad Prism 8 using One-way ANOVA. **p ≤ 0.01, ***p ≤ 0.001. Error bar represents mean ± SD.

Proteoglycans are major components of the extracellular matrix (ECM) and comprise a large, heterogeneous group, including heparin sulfate proteoglycans (HSPGs) and chondroitin sulfate proteoglycans (CSPGs) (Xia et al., 2021). Accumulation of glypicans on the cell surface of COG4-SWS derived patient’s fibroblast, and COG4-G516R zebrafish mutant has been reported previously (Ferreira et al., 2018; Xia et al., 2021). To examine potential change in proteoglycan accumulation in the cell surface of the RPE1 cells expressing different COG4 proteins, we used an antibody to HS-stubs (clone 3G10). These antibodies recognize the 3G10 neo-epitope (unsaturated uronic acid) exposed by digestion with heparinase III. WB revealed a significant increase in core proteins of HSPGs accumulation on the cell surface of both COG4-G516R and COG4-R729W mutant cell lines. (Figures 7A,B).

### Abnormal Secretion of SIL1 and ERGIC53 in COG4-G516R Mutant Cell Line

**FIGURE 8:**
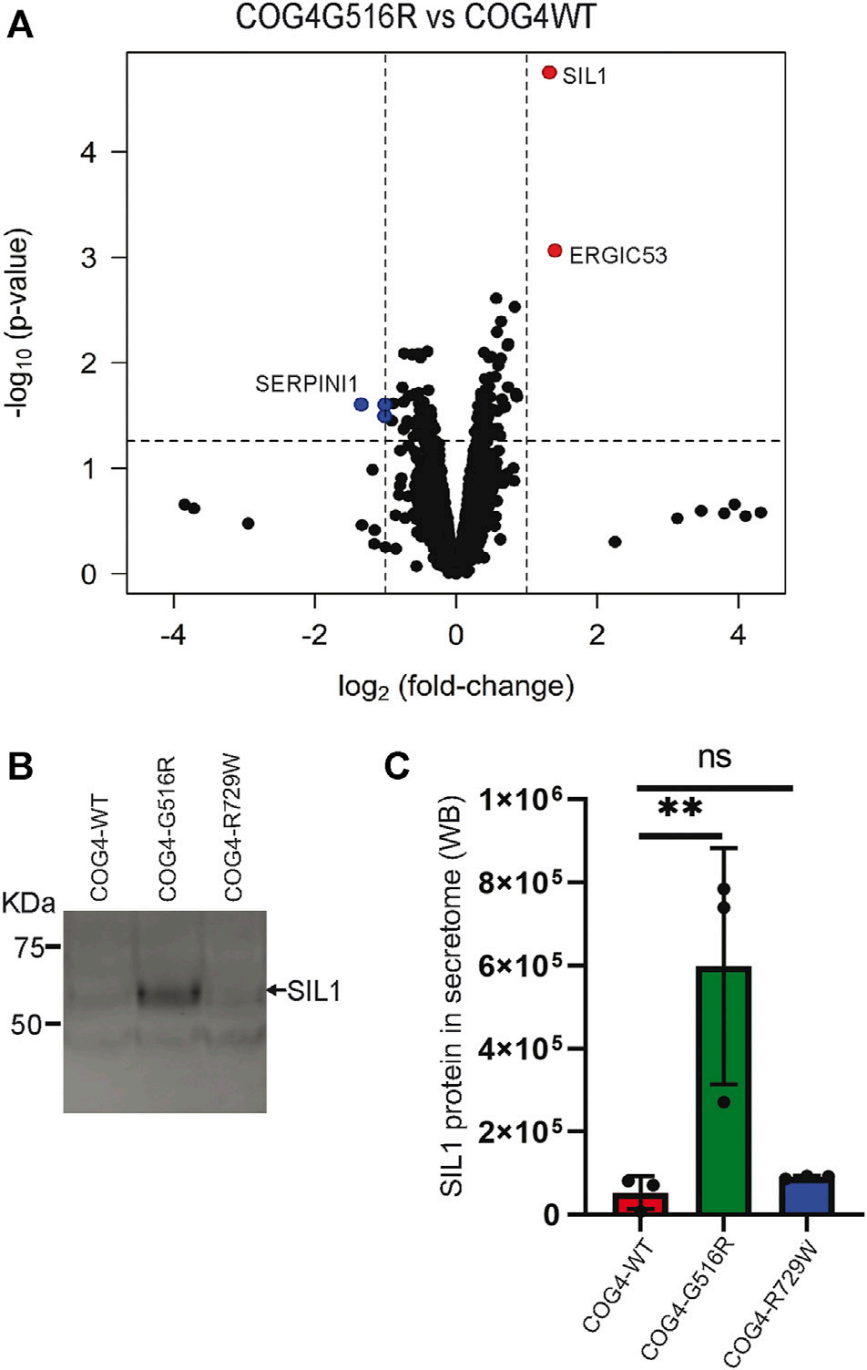
Abnormal secretion of SIL1 and ERGIC53 from COG4-G516R mutant cell line. **(A)** Volcano plot obtained from quantitative mass spectrometry analysis of protein secretome from RPE1 rescued COG4-WT-3myc and COG4-G516R-3myc cell lines (x-axis represents the log2 of the fold change, the y-axis represents the negative decade logarithm of the significance value). Red dots and blue dots represent proteins that are significantly upregulated and downregulated **(B)** Western blot analysis of the secretome from three rescued cell lines respectively and stained with the SIL1 antibody. **(C)** Quantitative analysis of secreted SIL1 protein level. Statistical significance was calculated by GraphPad Prism 8 using One-way ANOVA. **p ≤ 0.01 and non-significant (ns) *p* > 0.05. Error bar represents mean ± SD.

COG complex is regulating intra-Golgi trafficking (Blackburn et al., 2019), and we have shown previously that COG4 depletion is significantly affecting a spectrum of secretory proteins (D'Souza et al., 2019). To test if the expression of COG4-G516R changes the spectrum of secretory proteins, we performed an unbiased analysis of the total protein secretome from RPE1 cells using a quantitative TMT mass spectrometry approach. Analysis revealed 3,404 protein signatures in both control and COG4-G516R samples. Surprisingly, more than 99% of detected proteins were released from both cell lines to the same level. Secretion of three proteins (TMCO4, S100A-1, and SERPINI1) was significantly reduced in COG4-G516R mutant, while secretion of SIL1 and LMAN1/ERGIC53 was significantly increased (Figure 8A). The most prominent ( >10 times) increase in G516R secretome was detected for the ER luminal glycoprotein SIL1. WB analysis of the secretomes from all rescued cell lines confirmed a significant increase in the secretion of SIL1 protein by COG4-G516R and revealed that SIL1 secretion did not occur in COG4-R729W cells (Figures 8B,C).

## Discussion

We have developed a strategy for a cell-based model to compare different human mutations in the COG complex on the same genetic background. In this setup, we utilize a non-cancerous cell line in which the COG subunit is first deleted using the CRISPR/Cas9 approach and then stably restored by virus-assisted insertion of COG subunit wild-type CDG variants driven by the endogenous promoter. We have previously developed and characterized a complete set of HEK293T cells with a complete knockout of each of the COG complex subunits (Blackburn and Lupashin, 2016). To test this novel strategy, we created RPE1 and HEK293T cells expressing COG4 mutations COG4-G516R (Saul-Wilson syndrome) and COG4-R729W (COG-CDG type IIj) under the endogenous COG4 promoter. Saul-Wilson syndrome due to COG4 G516R mutation results in a rare skeletal dysplasia which is not categorized as CDG as defined in COG4 mutation R729W (Ferreira et al., 2018; D'Souza et al., 2020). It has been suggested that COG4-R729W mutation presents a partial loss of COG4 function, whereas the G516R mutation shows an abnormal gain of COG4 function (Reynders et al., 2009; Ferreira et al., 2018). Patient fibroblasts are traditionally used to reveal cellular defects associated with COG complex human mutations (Steet and Kornfeld, 2006, 7; Reynders et al., 2009; Ferreira et al., 2018). The major limitation of fibroblasts-based studies is potential heterogeneity resulting from a diverse genetic background of the patients. Another limitation is linked to the fibroblast’s cell physiology which may not reveal specific defects manifested in nervous, ocular, bone, and other tissues severely affected in COG patients (D'Souza et al., 2020). The presented cellular model mostly overcomes these limitations by providing tissue-specific uniform genetic background, allowing side-by-side comparison of different COG mutations.

Comparison of COG4 G516R and R729W variants expressed in both RPE1 and HEK293T cells under COG4 promoter revealed that both mutant proteins are properly Golgi localized and expressed at the wild-type level. Moreover, we did not find any significant disruption of Golgi structure resulting from the expression of these COG mutants as previously observed in some of the patients’ fibroblasts (Reynders et al., 2009; Ferreira et al., 2018), indicating that both mutants enter the COG complex and capable of restoring the majority of cellular defects observed in cells completely depleted for COG4 protein. Our finding agrees with the recent study of COG4-G516R mutation in the worm model (Zafra et al., 2021), but is different from disrupted and less rigid Golgi stack found in R729W patients’ fibroblasts (Reynders et al., 2009) and “collapsed” Golgi stacks observed in G516R patients’ fibroblasts (Ferreira et al., 2018). Our results indicate that structural Golgi changes observed in some patients’ fibroblasts are cell-type dependent and do not represent the prevailing cellular phenotype associated with G516R and R729W mutations. On the other hand, we did confirm O-glycosylated defects associated with G516R mutation and N-glycosylated defects associated with R729W mutation. The N-glycosylation defects have been reported in HeLa cells bearing R729W mutation (Richardson et al., 2009). Specific defect in N-glycosylation observed in cells bearing R729W mutation may be due to the loss of the subset of intra-Golgi recycling vesicles carrying MGAT1, medial Golgi enzyme responsible for the addition of the first GlcNAc residue in the Golgi (Kumar et al., 1990). The exact mechanism of MGAT1 recycling is still an enigma, but we have shown previously that this protein is mislocalized in cells depleted for COG4 proteins (Pokrovskaya et al., 2011). It is less clear why cells bearing G516R mutation are preferentially deficient in O-glycosylation. The HPA lectin recognizes exposed α-N-acetyl-d-galactosamine residue (Irimura et al., 1981) in immature O-glycosylated proteins. The staining for this lectin is significantly increased in cells deficient for the Golgi-to-ER recycling of polypeptide N-acetylgalactosaminyl transferases (GalNac-Ts) (Gill et al., 2010). One possibility is that the COG4 G516R mutant is defective in recognition of a subset of vesicles recycling GalNac-Ts. Depletion of COG complex subunits results in rapid accumulation of CCD (COG complex dependent) vesicles (Zolov and Lupashin, 2005; Shestakova et al., 2006); future investigation of CCD vesicle population should reveal distinct subsets regulated by different COG subunits and arrangements.

Ocular involvement has been reported in 60% of CDG with a pure O-glycosylation defect (Francisco et al., 2018). G516R mutated patients also have ocular phenotypes, which are well aligned with our findings revealing O-glycosylation defects. On the other hand, different N-glycosylation defects include phenotypes such as intellectual disability, speech disorder, and abnormal gait (Wolthuis et al., 2014), similar to our findings showing N-glycosylation defects in the R729W mutated cell line. We also confirmed the accumulation of heparin sulfate proteoglycans on the surface of cells expressing COG4-G516R mutation (Xia et al., 2021); however, our studies revealed an even greater accumulation of HSPGs in cells expressing the COG4-R729W variant. This result indicates that HSPGs accumulation alone may not be sufficient to explain the unique SWS phenotype associated with COG4 G516R mutation. Moreover, it was recently reported that HSPG modifications are severely altered in HEK293T cells depleted for six different COG complex subunits (Adusmalli et al., 2021).

Our previous work revealed an abnormal protein secretion in COG4 deficient cells (Blackburn et al., 2018). This observation prompted us to investigate the protein secretome of COG4-G516R expressing cells. Unbiased quantitative mass-spectrometry analysis revealed no difference in secretion of 99.9% of detected proteins. Importantly, two proteins, SIL1 and ERGIC53 were released from COG4-G516R cells at a much higher level as compared to wild-type cells or cells expressing COG4-R729W. SIL1/BAP is the ER-resident soluble glycoprotein that is usually not secreted from cells. Its ER localization is thought to be mediated by tetra-peptide (KELR) at the C terminus (Chung et al., 2002), but the exact mode of SIL1 intracellular retention has not been studied in detail. SIL1 functions as a nucleotide exchange factor and co-chaperone for ER lumenal chaperone HSPA5/GRP78/BIP which is required for protein translocation and folding (Ichhaporia and Hendershot, 2021). Our proteomic analysis did not detect abnormal secretion of HSPA5, or any other ER-resident proteins retained by the KDEL-based mechanism. Interestingly, SIL1 overexpression causes the disturbance of the Golgi structure (Labisch et al., 2018), indicating a potential link between SIL1 and COG trafficking machinery.

SIL1 is ubiquitously expressed in different tissues but at a different level. Though the co-chaperone mutation might affect highly secretory tissues, such as the kidney, liver, placenta, and plasma cells but SIL1 mutation significantly affect the neuronal cell, lens epithelial cell, and skeletal muscle (Krieger et al., 2013; Ichhaporia et al., 2015; Ichhaporia and Hendershot, 2021). Interestingly, the Saul-Wilson syndrome presents skeletal and ocular defects similar to SIL1 mutation (loss of function) phenotypes. Therefore, SIL1 mutation could be the major contributing factor to Saul-Wilson syndrome. ERGIC53/LMAN1 is a transmembrane glycoprotein that shuttles between ER and the Golgi apparatus and is the hallmark of the ERGIC/IC compartment (Zhang et al., 2009). It acts as a secondary quality control mechanism complementary to the ER folding machinery (Zhang et al., 2009). Importantly, ERGIC53 is a mannose-binding lectin (Baines and Zhang, 2007), and therefore ERGIC53 abnormal release from COG4-G516R cells may be connected to the elevated release of SIL1 glycoprotein.

Two commonly used non-cancerous diploid (RPE1) and triploid (HEK293T) cell lines were utilized for our initial studies. We realized that the choice of cell lines could limit our understanding of skeletal and liver defects common for COG-CDG patients. To overcome these potential limitations, similar experiments with skeletal hFOB and human iPSC cell lines were recently initiated. A more sophisticated alternative to our approach is the use of CRISPR/Cas9 gene-editing strategy to introduce known COG-CDG mutation directly into the genome (Hsu et al., 2014) of a chosen cell line. This strategy avoids potential off-target effects and may be more suitable for mutations that affect the splicing of COG subunits (Hsu et al., 2014). At the same time, the correct bi-allelic introduction of desired mutations is far more complicated as compared to the strategy presented in this work and would require single-cell cloning, introducing potential genome heterogeneity. Moreover, CRISPR editing requires homolog-driven repair (HDR). It has been reported previously that the low efficiency of HDR decreases its utility for precise gene editing, and to increase the HDR efficiency, NHEJ (non-homologous end joining) needs to be inhibited (Uddin et al., 2020).

In the future, we aim to extend this strategy to other COG mutations and expand the model system to the bone cell line hFOB as well as to stem cells. We also plan to characterize Golgi and plasma membrane glycoproteins and glycolipids using quantitative mass-spectrometry techniques. We hope that this approach will help in the identification of key problems in different COG mutants and ultimately will help in the treatment strategy for COG-CDG patients.

## Data Availability

The datasets presented in this study can be found in online repositories. The names of the repository/repositories and accession number(s) can be found below: Data are available via ProteomeXchange with identifier PXD027202.
