# Supplementary figures and images for: Development and Initial Characterization of Cellular Models for COG Complex-Related CDG-II Diseases
Source: Front Genet. 2021 Sep 17;12:733048. doi: 10.3389/fgene.2021.733048 (PMC8484713; doi:10.3389/fgene.2021.733048)

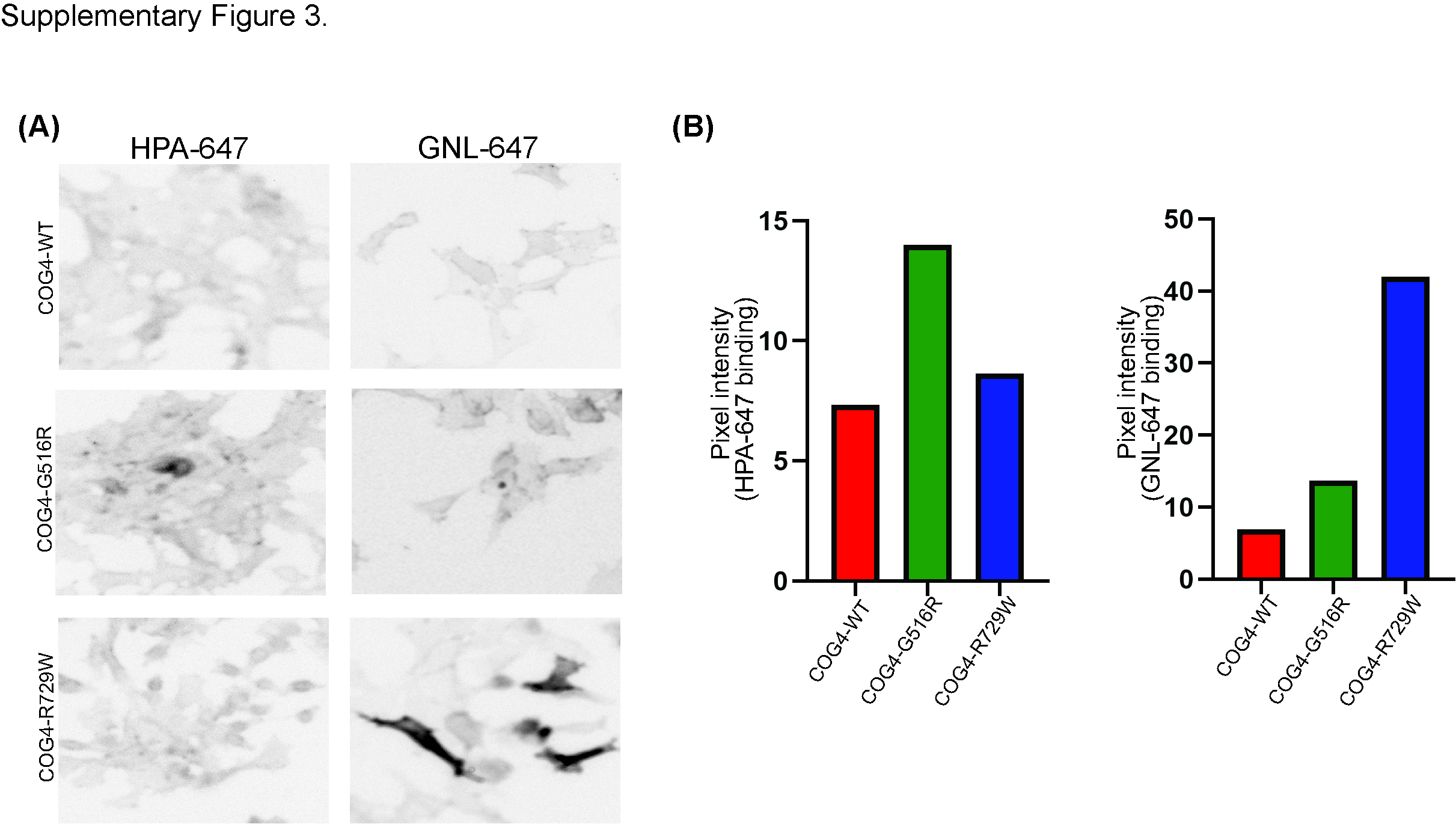

Supplement: Supplementary file 1 [file Image3.TIF]

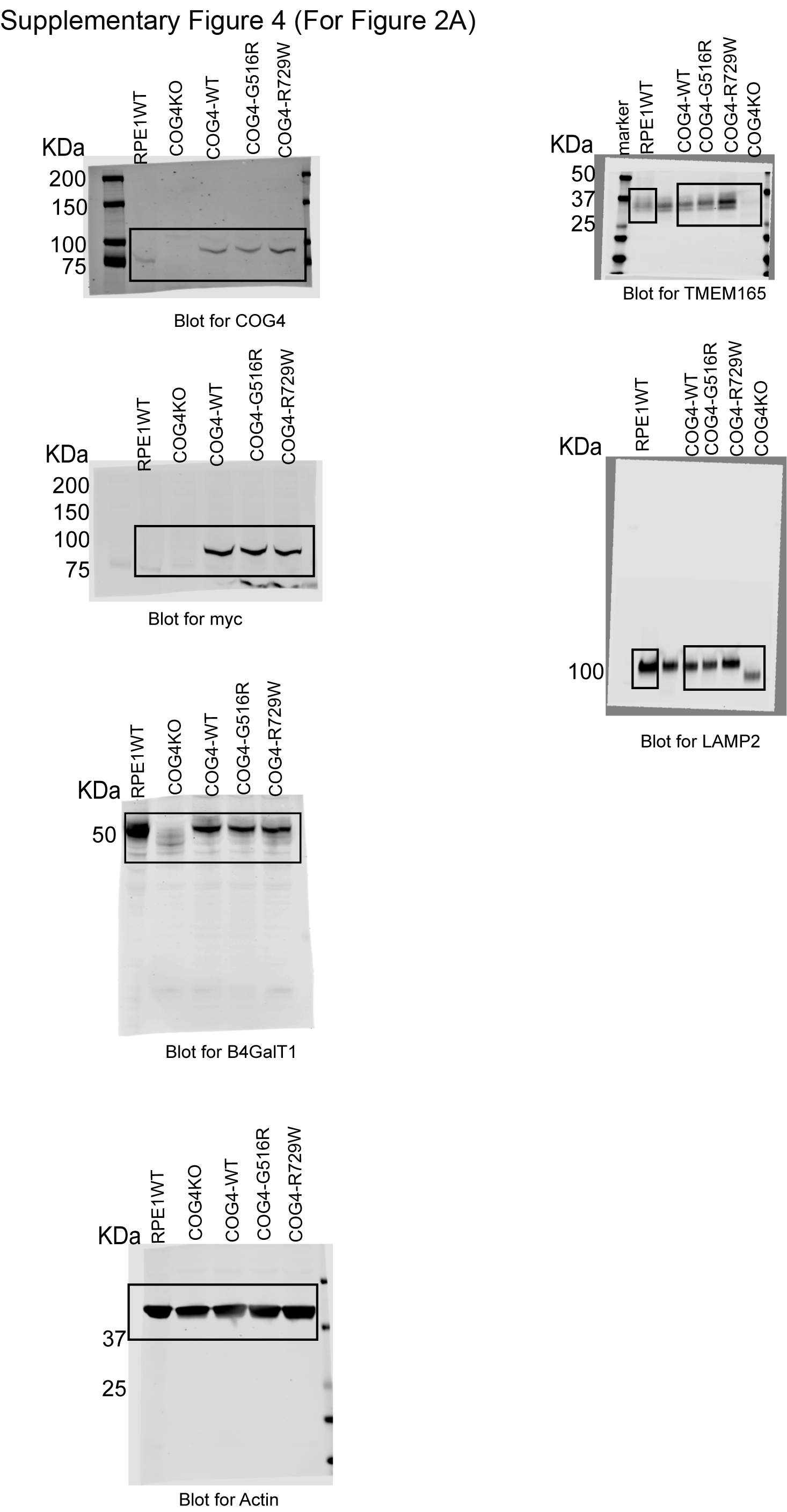

Supplement: Supplementary file 2 [file DataSheet1.ZIP › Supplementary 4 (Figure 2A).tif]

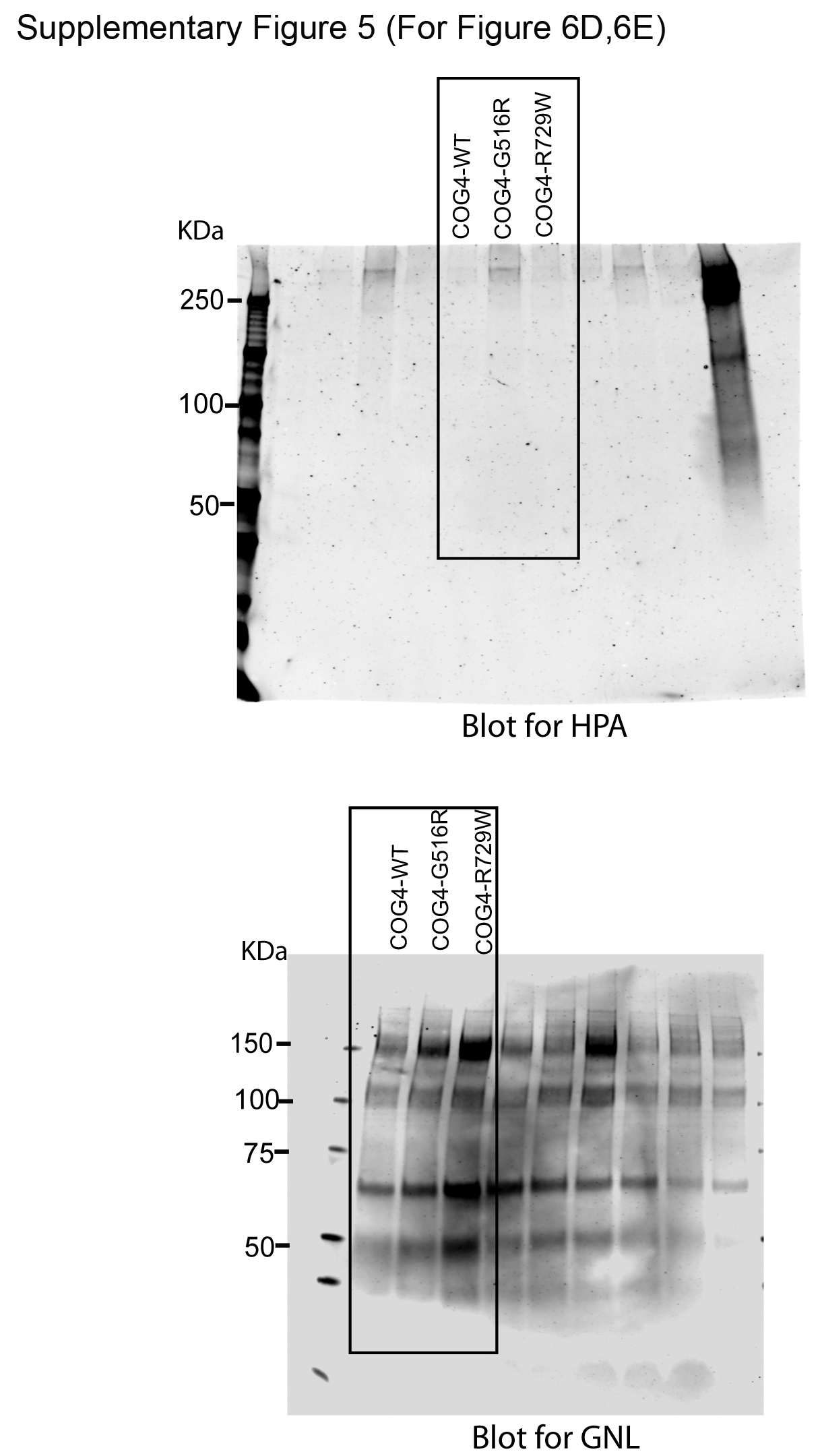

Supplement: Supplementary file 2 [file DataSheet1.ZIP › Supplementary 5 (for Figure 6D, 6E).tif]

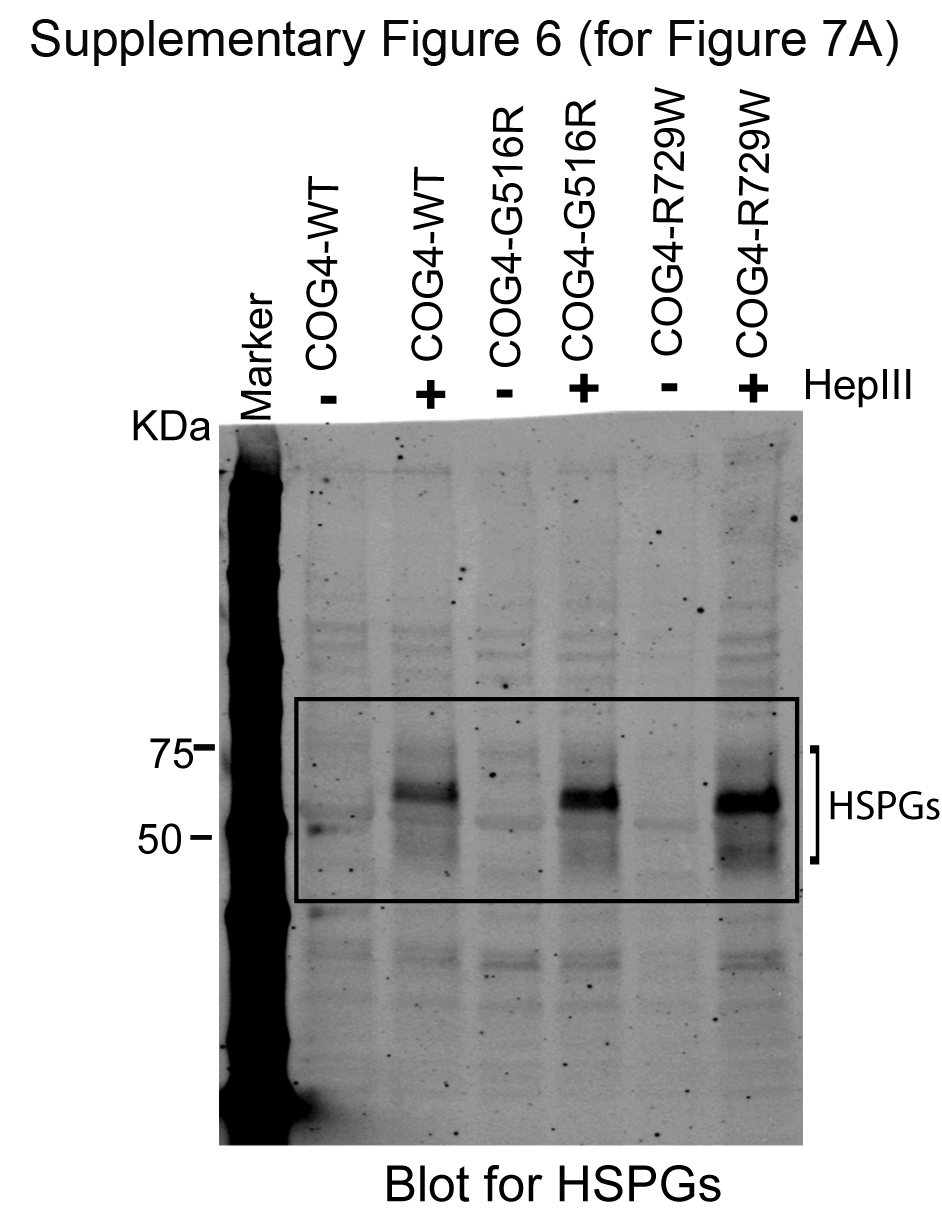

Supplement: Supplementary file 2 [file DataSheet1.ZIP › Supplementary 6 (for Figure 7A).tif]

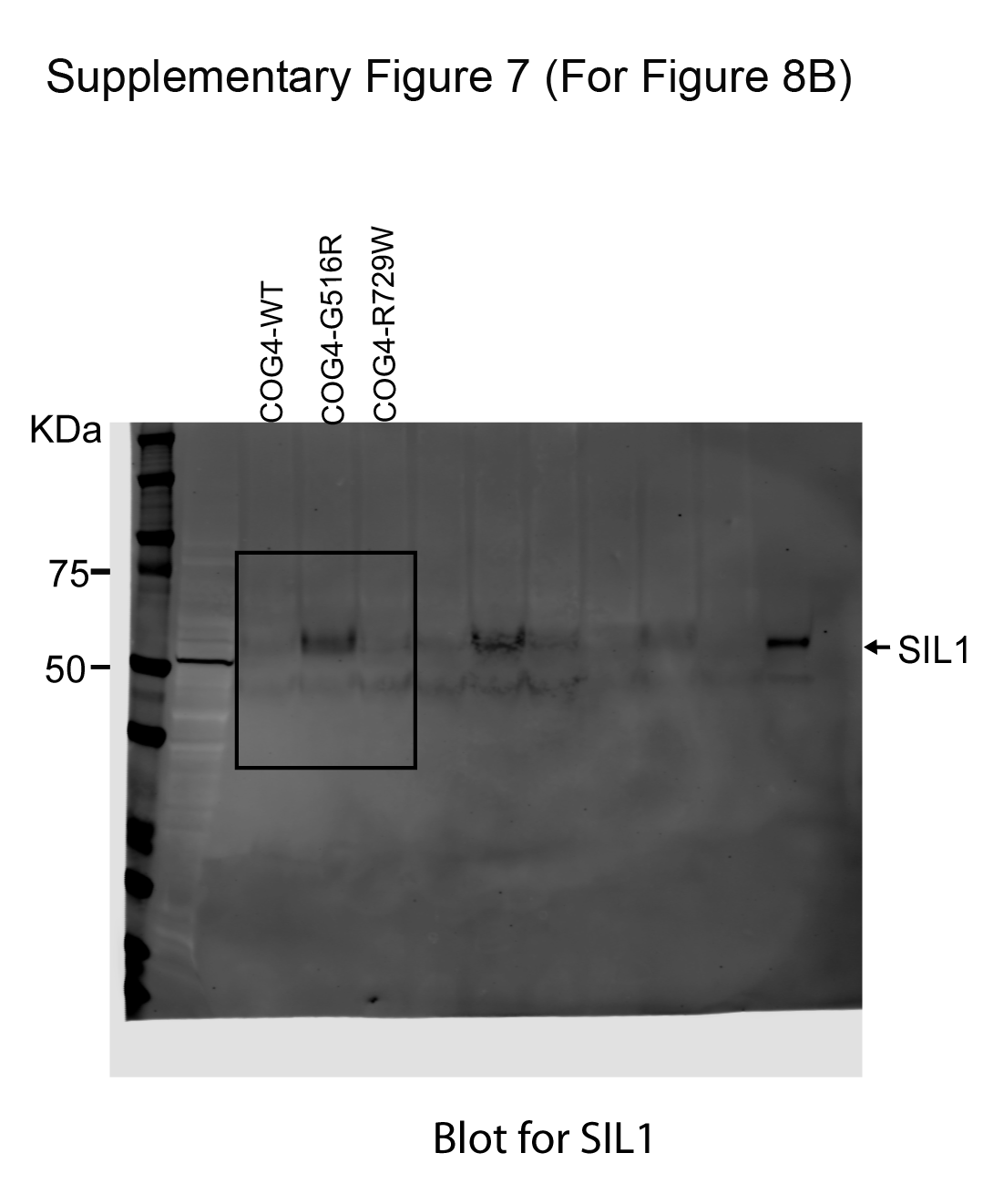

Supplement: Supplementary file 2 [file DataSheet1.ZIP › Supplementary 7 (for Figure 8B).tif]

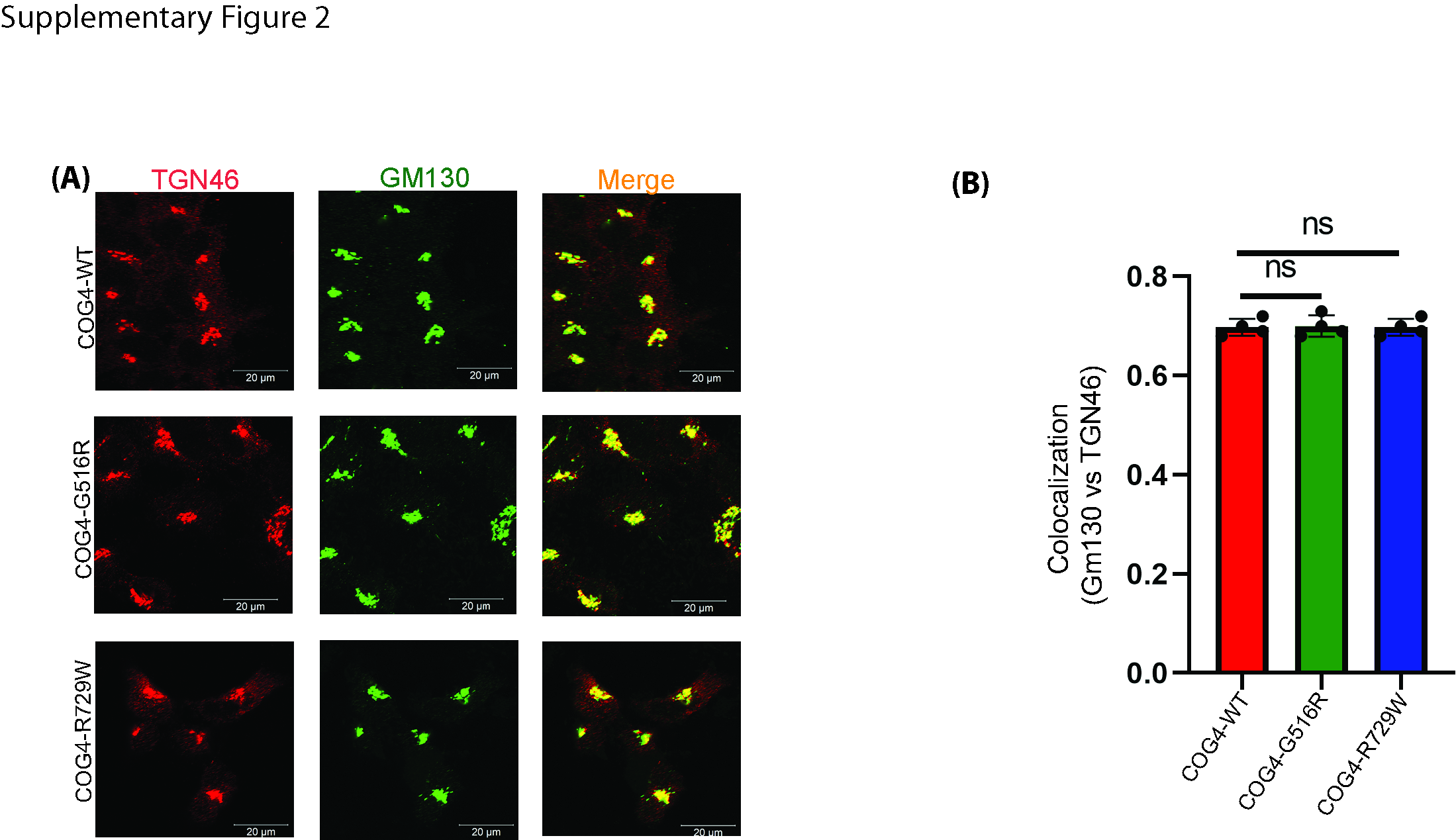

Supplement: Supplementary file 3 [file Image2.TIF]

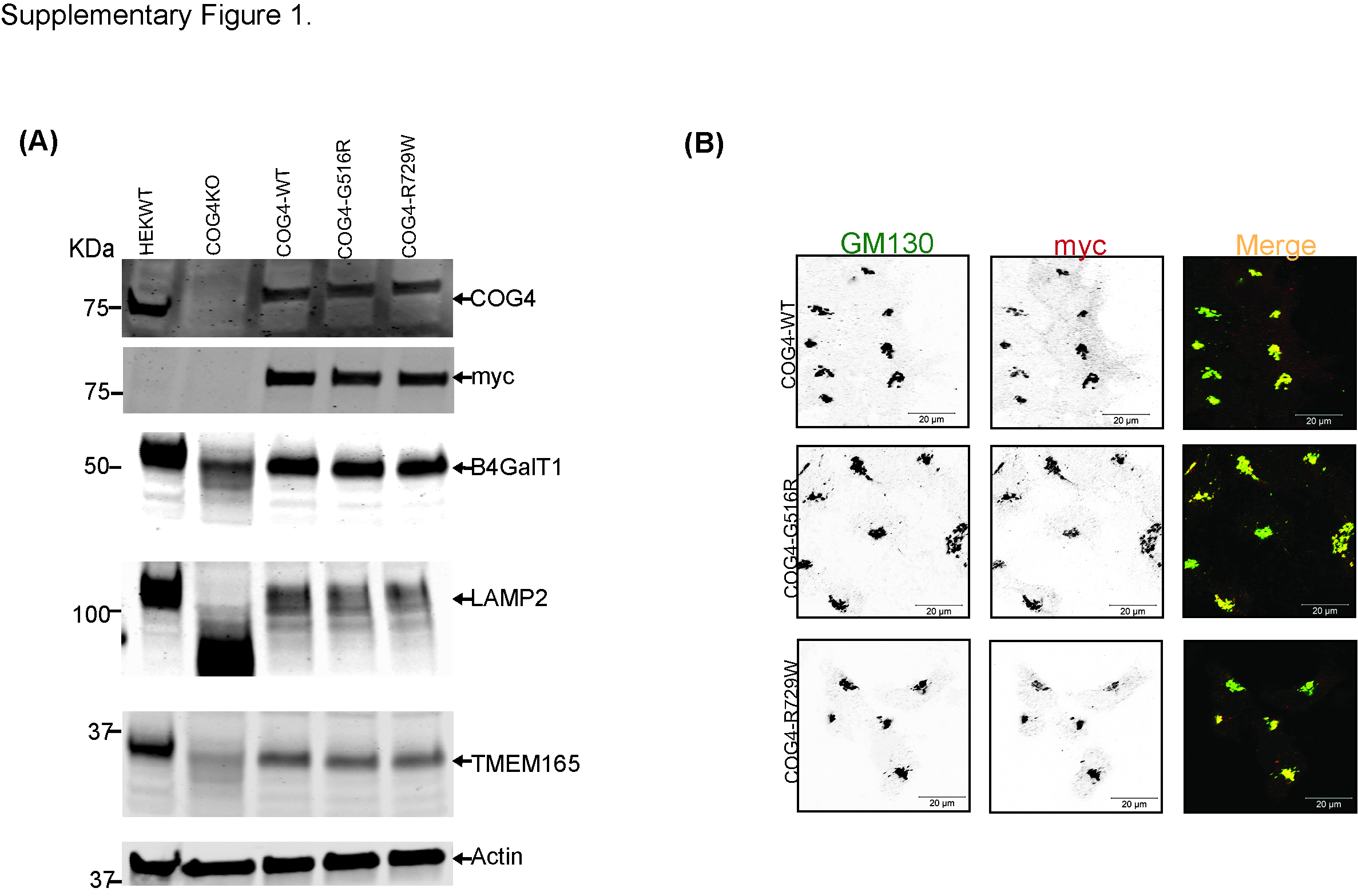

Supplement: Supplementary file 4 [file Image1.TIF]
