# Supplementary material for: Development and Initial Characterization of Cellular Models for COG Complex-Related CDG-II Diseases
Source: Front Genet. 2021 Sep 17;12:733048. doi: 10.3389/fgene.2021.733048 (PMC8484713; doi:10.3389/fgene.2021.733048)
Supplement: Supplementary file 5 [file Table2.DOCX]

**Supplementary Table 2: List of antibodies**

| **Antibody** | **Source/Catalog #** | **Species** | **Dilution (WB)** | **Dilution (IF)** |
| --- | --- | --- | --- | --- |
| Giantin | (Covance PRB-114C | Rabbit | - | 1:2000 |
| GM130 | CalBiochem, CB1008 | Rabbit | - | 1:2000 |
| Β-actin | Sigma, A5541 | Mouse | 1:2000 | - |
| TGN46 | Bio-Rad AHP500G | Sheep | 1:2000 | 1:500 |
| LAMP2 | DSHB, H4B4 | Mouse | 1:2000 | - |
| TMEM165 | Sigma HPA038299 | Rabbit | 1:2000 | - |
| B4GalT1 | R&D Systems AF-3609 | Goat | 1:500 | 1:500 |
| Myc-tag | Cell Signaling 9B11 | Mouse | 1:2000 | 1:2000 |
| COG4 (C terminal) | Sigma SAB4200469 | Rabbit | 1:1000 | - |
| ERGIC53 | Enzo OTI1A8 | Mouse | - | 1:1000 |
| GS15 | BD 610961 | Mouse | - | 1:400 |
| SIL1 | Proteintech 24110-1-AP | Rabbit | 1:1000 | - |
| ∆Heparan sulfate (F69-3G10) | Amsbio 370260-s | Mouse | 1:500 | - |
| IRDye 800 anti-Goat | LiCOR/926-32214 | Donkey | 1:20000 | - |
| IRDye 800 anti-Mouse | LiCOR/ 5-32210 | Goat | 1:20000 | - |
| Alexa Flour 647 anti-Goat | Jackson Immuno Research/705605-147 | Donkey | 1:4000 | 1:500 |
| Alexa Fluor 647 anti-Sheep | Jackson Immuno Research/705605-147 | Donkey | 1:4000 | 1:500 |
| Anti-Rabbit Cy3 | Jackson Immuno Research/705605-152 | Donkey | 1:4000 | 1:500 |
| Anti-Mouse Cy3 | Jackson Immuno Research/705605-151 | Donkey | 1:4000 | 1:500 |
| Alexa Fluor 488 anti-Rabbit | Jackson Immuno Research/705605-151 | Donkey | - | 1:500 |
| Alexa Fluor 647 anti-mouse | Jackson Immuno Research/705605-151 | Donkey | 1:4000 | 1:500 |
| Alexa Fluor 647 anti-rabbit | Jackson Immuno Research/705605-152 | Donkey | 1:4000 | 1:500 |
